# The emerging roles of microbiome and short-chain fatty acids in the pathogenesis of bronchopulmonary dysplasia

**DOI:** 10.3389/fcimb.2024.1434687

**Published:** 2024-09-20

**Authors:** Yuan Gao, Kaixuan Wang, Zupan Lin, Shujing Cai, Aohui Peng, Le He, Hui Qi, Zhigang Jin, Xubo Qian

**Affiliations:** ^1^ Neonatal Intensive Care Unit (NICU), Jinhua Maternal and Child Health Care Hospital, Jinhua, China; ^2^ Department of Pediatrics, Affiliated Jinhua Hospital, Zhejiang University School of Medicine, Jinhua, China; ^3^ College of Life Sciences, Zhejiang Normal University, Jinhua, China; ^4^ Department of Pediatrics, Jinhua Hospital of TCM Affiliated to Zhejiang University of Traditional Chinese Medicine, Jinhua, China; ^5^ China National Clinical Research Center of Respiratory Diseases, Respiratory Department, Beijing Children’s Hospital, Capital Medical University, National Center for Children’s Health, Beijing, China; ^6^ Central Laboratory, Affiliated Jinhua Hospital, Zhejiang University School of Medicine, Jinhua, China

**Keywords:** bronchopulmonary dysplasia, microbiota, microbiome, short-chain fatty acids, gut-lung axis, immune modulation, barrier function

## Abstract

Bronchopulmonary dysplasia (BPD) is a chronic lung disease that affects premature infants and leads to long-term pulmonary complications. The pathogenesis of BPD has not been fully elucidated yet. In recent years, the microbiome and its metabolites, especially short-chain fatty acids (SCFAs), in the gut and lungs have been demonstrated to be involved in the development and progression of the disease. This review aims to summarize the current knowledge on the potential involvement of the microbiome and SCFAs, especially the latter, in the development and progression of BPD. First, we introduce the gut-lung axis, the production and functions of SCFAs, and the role of SCFAs in lung health and diseases. We then discuss the evidence supporting the involvement of the microbiome and SCFAs in BPD. Finally, we elaborate on the potential mechanisms of the microbiome and SCFAs in BPD, including immune modulation, epigenetic regulation, enhancement of barrier function, and modulation of surfactant production and the gut microbiome. This review could advance our understanding of the microbiome and SCFAs in the pathogenesis of BPD, which also helps identify new therapeutic targets and facilitate new drug development.

## 1 Introduction

Bronchopulmonary dysplasia (BPD), also called chronic lung disease of prematurity, is a chronic lung disease that primarily affects premature infants. It is characterized by lung injury, inflammation, oxidative stress, pulmonary vascular abnormalities, and impaired lung development (Bush et al., 2023). BPD has a prevalence of approximately 70.0% in infants born before 28 weeks of gestation and 79.4% in infants born before 26 weeks of gestation (Zhu et al., 2021; Bell et al., 2022). This disease is a significant cause of morbidity and mortality in the neonatal population, despite advances in neonatal care. The burden of BPD extends beyond the neonatal period, with affected infants experiencing long-term respiratory and neurodevelopmental sequelae (Bell et al., 2022). The economic burden of BPD is also substantial (Alvarez-Fuente et al., 2017; Lapcharoensap et al., 2020). For example, the median hospitalization cost is $377,871 per infant with BPD in the first year of life, which is more than twice that of other children (Lapcharoensap et al., 2020).

The pathogenesis of BPD is not fully understood. However, several risk factors have been associated with the development of BPD, including chorioamnionitis, intrauterine growth restriction, prematurity, mechanical ventilation, oxygen toxicity, and infection (Dankhara et al., 2023; Deng et al., 2023; Salimi et al., 2022). In recent years, microbiome and its metabolites in the gut and lungs have emerged as potential key players in the pathogenesis of BPD (Frazer et al., 2022; Boel et al., 2024; Shen et al., 2024). The microbiome, along with its metabolites, immune cells, and other molecules in the gut, can influence respiratory health and vice versa; this bidirectional communication is referred to as the gut-lung axis (Ozcam and Lynch, 2024). Short-chain fatty acids (SCFAs), the main metabolites of microbiome, are the end products of microbial fermentation of dietary fiber in the gut, which have been shown to exert numerous beneficial effects on host physiology (Mann et al., 2024). Dysbiosis of microbiome, which disrupts SCFA production and metabolism, has been linked to the development of BPD (Campbell et al., 2024; Shen et al., 2024).

In this review, we comprehensively evaluate the existing literature on the key roles of microbiome and SCFAs in BPD, focusing on the gut-lung axis, evidence supporting their involvement, and potential mechanisms. The potential mechanisms include immune modulation, epigenetic regulation, enhancement of barrier function, and modulation of surfactant production and the gut microbiome. Understanding the roles of SCFAs in BPD could pave the way for novel therapeutic strategies targeting the gut microbiome and SCFA, potentially preventing or mitigating the development of BPD in preterm infants (Sorbara and Pamer, 2022; Shen et al., 2024).

## 2 Gut-lung axis

During the early stages of embryonic development, both the gut and lungs originate from the same primitive foregut (Wypych et al., 2019). After birth, these organs serve as crucial mucosal barriers, separating deeper tissues from the external environment. The two organs have different cell types, distinct compositions of mucosa, and different densities of immune cells, while they share several similarities: both are exposed to the external environment, have large surface areas and a mucus barrier, facilitate the passage of vital nutrients in the gut and gases in the lungs, prevent the translocation of luminal antigens and pathogens into the host circulation, produce secretory immunoglobulin A (IgA), and possess their own unique microbiome (Liang et al., 2022; Barreto and Gordo, 2023; Natalini et al., 2023). The microbiome that resides within the two organs of healthy humans comprises bacteria, archaea, viruses, and fungi (Li et al., 2024). Additionally, protozoans and metazoans also inhabit the gut of healthy humans (Ianiro et al., 2022). In healthy individuals, the lung microbiome exhibits a low biomass and is primarily dominated by oral commensals such as *Streptococcus*, *Prevotella*, and *Veillonella*, which is characterized by frequent changes due to microbial immigration and clearance, rather than a fixed composition (Natalini et al., 2023). In this review, we mainly focus on bacteria and their metabolites, SCFAs.

**Figure 1:**
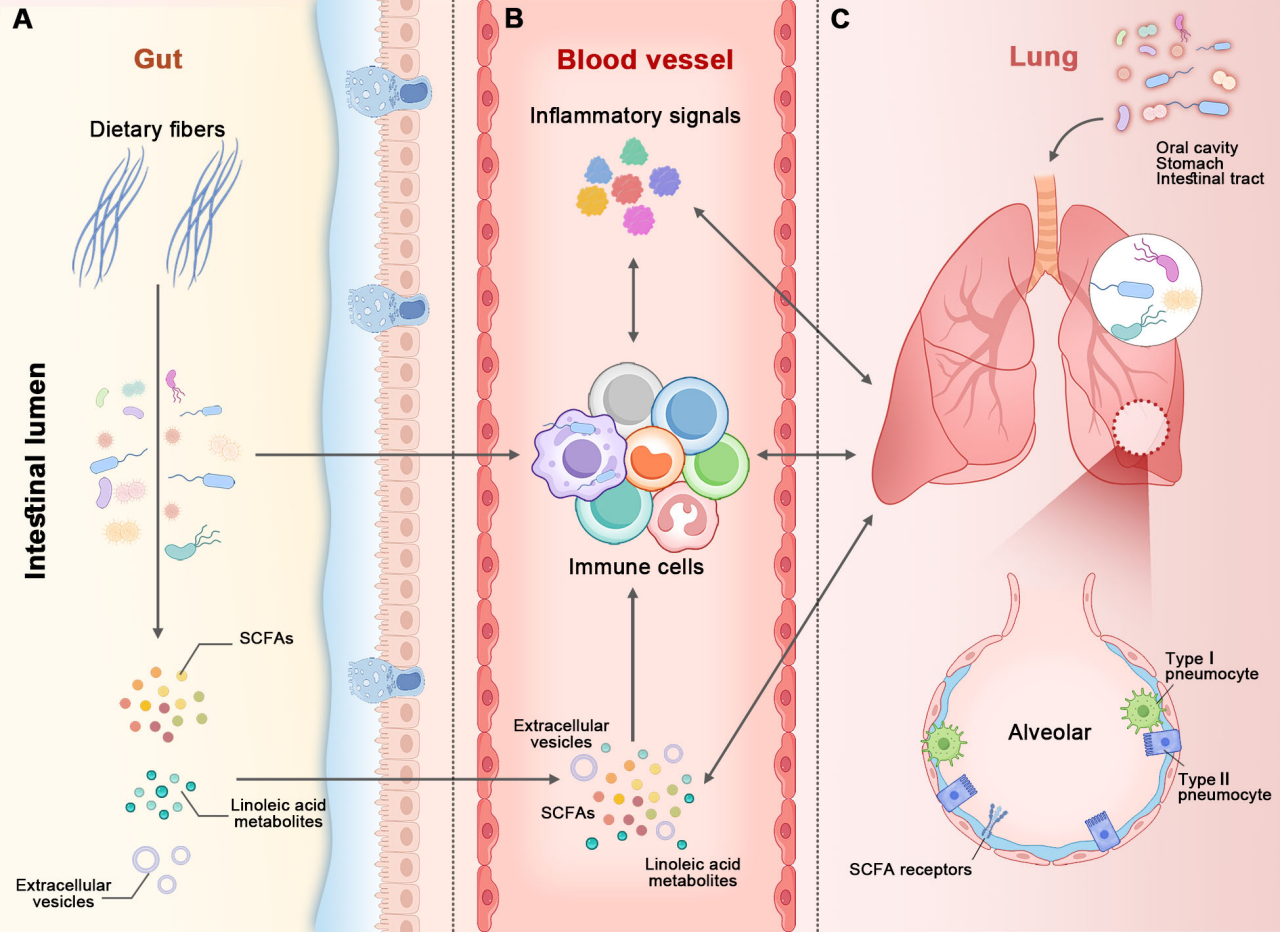
The figure illustrates the gut-lung axis. The microbiome, along with its metabolites, immune cells, and other molecules, acts as bidirectional communicators between the gut and lungs. There are at least five pathways connecting the two organs: ⑴ The intestinal lumen (in panel **A**) and airways (in panel **C**) each possess their own microbiome. The gut microbiome (in panel **A**) can translocate to the lungs (in panel **C**) via aspiration of oral or gastric contents (at the top of panel **C**), immune cells, and the bloodstream (in panel **B**). ⑵ Short-chain fatty acids (SCFAs) derived from the gut microbiome (in panel **A**) enter the bloodstream and then make their way to the lungs, and vice versa. ⑶ Extracellular vesicles produced in the gut are transported to the lungs via the bloodstream, and vice versa. ⑷ Immune cells and molecules migrate or traverse between the two organs. ⑸ Inflammatory signals from the two organs influence each another. All of these mechanisms can be observed in healthy populations, with the exception of microbiome translocation.

The two organs interact with each other, and the bidirectional communication between them is referred to as the gut-lung axis (Ozcam and Lynch, 2024) (**Figure 1**). As the important communicators in the gut-lung axis, the microbiome and its metabolites play key roles in maintaining the health of the two organs. The mechanisms of interaction between the gut and lungs remains incompletely understood, yet several pathways have been proposed (**Figure 1**). Firstly, the microbiome, encompassing bacteria, fungi, and viruses, resides in both organs (Li et al., 2024). These microbial communities can directly or indirectly interact, thereby impacting each other’s composition and function. For instance, the gut microbiome can translocate into the lungs (Wheatley et al., 2022). This translocation can occur through various mechanisms, including aspiration of oral or gastric contents, migration of immune cells carrying gut microbes, and dissemination of bacteria through the bloodstream (Wheatley et al., 2022; Yang et al., 2022). Once the translocation occurs, these gut-derived microbes can potentially influence lung health and immune responses. It is important to note that the translocation is infrequent in healthy populations. Secondly, the metabolites derived from the gut, such as SCFAs and linoleic acid, can have systemic effects and influence lung health (Dai et al., 2024; Ozcam and Lynch, 2024). These metabolites have the capacity to modulate immune responses, inflammation, and even the functionality of lung cells, acting as a key link between them. Thirdly, extracellular vesicles are small, membrane-bound particles that are released by cells and microbes into their environment. They can carry a wide range of microbial pathogen-associated molecular patterns and other molecules, including cell wall components, proteins, lipids, nucleic acids, and metabolites. The gut is an important site for producing extracellular vesicles that are transported to the lungs, where they have diverse functions such as intercellular communication, immune modulation, and tissue repair (Iannotta et al., 2024; Ozcam and Lynch, 2024). Fourthly, the immune system serves as a crucial link between the gut and lungs. Immune cells and molecules possess the ability to migrate between these organs, thereby regulating immune responses and inflammation in a reciprocal manner (Ni et al., 2023). Lastly, inflammatory molecules emerge as a pivotal factor within the gut-lung axis. Inflammatory signals such as cytokines can traverse between the two organs, thereby triggering immune activation and conceivably influencing each other (Enaud et al., 2020). All the mechanisms of interaction between the gut and lungs occur in patients. However, not all of these mechanisms can be observed in healthy populations, such as the translocation of gut microbiome into other parts of the body.

## 3 SCFAs: production and functions

SCFAs are volatile fatty acids with six or fewer carbon atoms. They are primarily produced in the colon through the bacterial fermentation of dietary fiber, which are complex carbohydrates that humans cannot digest. The lungs might not have the ability to produce SCFAs, and the SCFAs present in the lungs are likely derived from the gut (Ozcam and Lynch, 2024). The main SCFAs produced are acetate, propionate, and butyrate. The production of SCFAs is largely dependent on the composition of the gut microbiome and the type of dietary fiber consumed (Xiong et al., 2022). Different bacterial species have different capacities to ferment dietary fiber and produce SCFAs (Kamareddine et al., 2020; de Vos et al., 2022). Therefore, a diverse and balanced gut microbiome, combined with a diet rich in various types of fiber, is essential for optimal SCFA production. SCFAs play pivotal roles in sustaining the physiological functions of the body. These compounds actively contribute to various processes, including energy metabolism, acting as signaling molecules, facilitating the intricate gut-brain and gut-lung axes, immunomodulation, and preservation of the mucosal barrier (de Vos et al., 2022).

Each SCFA has distinct roles in energy metabolism. Acetate, the most abundant SCFA, serves as a primary energy source for peripheral tissues and contributes to cholesterol synthesis. Moreover, acetate is a precursor molecule that is converted into acetyl-CoA, which is a key intermediate in the biosynthesis of cholesterol. However, in some contexts, acetate has inhibitory effects on cholesterol synthesis (Liu et al., 2019; Bakr and Farag, 2023). Propionate is involved in gluconeogenesis, the process of creating glucose in the liver, and it also inhibits cholesterol synthesis (Bakr and Farag, 2023). Butyrate, although less abundant, is the primary energy source for colonocytes and plays a critical role in maintaining the health and integrity of the gut barrier (Xiong et al., 2022; Bakr and Farag, 2023).

Beyond their roles in energy metabolism, SCFAs have emerged as key signaling molecules with systemic effects. They can bind to specific receptors on the surface, cytoplasm, or nucleus of cells, including G-protein-coupled receptor 41 (GPR41), GPR43, GPR109A, peroxisome proliferator-activated receptors γ, and aryl hydrocarbon receptor (Rekha et al., 2024). SCFAs bind to the GPRs to activate downstream pathways, such as the cAMP, MAPK, PI3K/Akt, and phospholipase C pathways (Rekha et al., 2024). These cascade reactions initiated by the binding of SCFAs to their respective GPRs often lead to transcriptional changes in the target cells.

Emerging evidence suggests that SCFAs can also influence other organs or systems. For example, SCFAs modulate brain function and behavior. This is often referred to as the gut-brain axis, highlighting the far-reaching effects of these gut-derived metabolites (Agirman et al., 2021; Hong et al., 2023; Ahrens et al., 2024). Recent research has highlighted the role of specific gut microbiome in modulating neurotransmitter production (Dohnalova et al., 2022), neuroinflammation (Seo et al., 2023), and even influencing mood and cognitive function (Chen et al., 2022; Hao et al., 2023). SCFAs are also the key communicators in the gut-lung axis (Verma et al., 2024).

In terms of the roles of SCFAs in immune regulation and maintaining barrier integrity, they will be detailed in the section “6 Potential mechanisms of microbiome and SCFAs in BPD”.

## 4 Role of SCFAs in lung health and disease

The roles of SCFAs in lung health and disease are multifaceted and are increasingly gaining attention in both basic and clinical research (Niu et al., 2023; Sikder et al., 2023; Zhou et al., 2023). The SCFAs are mainly produced in the gut, and they can be transported to the lungs via the bloodstream. These SCFAs in the two organs play significant roles in lung health and disease (Ashique et al., 2022).

There are several ways that SCFAs impact lung health. They can enhance the barrier function of the lung epithelium, similar to their actions in the gut, thus preventing the invasion of pathogens and reducing the risk of infections (Major et al., 2023; Chollet et al., 2024; Mann et al., 2024). They can also modulate the immune response in the lungs (Sikder et al., 2023; Mann et al., 2024), promote the resolution of inflammation and the clearance of pathogens, and prevent inflammation that can damage lung tissue (Ashique et al., 2022; Antunes et al., 2023). They have the similar effects in the gut (Mann et al., 2024). Additionally, emerging evidence suggests that SCFAs may have a role in surfactant production, which is another clue that SCFAs are associated with lung health and disease. For example, the receptors of SCFAs are found in alveolar macrophages and alveolar type II cells (Liu et al., 2021b), and the latter are the main cells producing the surfactant. SCFAs are involved in the maintenance of barrier integrity (Hu et al., 2024), immune regulation (Zheng et al., 2020; Sikder et al., 2023), and inflammation control (Shen et al., 2024) in the lungs, which can directly or indirectly influence the surfactant production. It should be noted that the evidence is limited, and further research is needed to investigate the role of SCFAs in the production of pulmonary surfactant.

An increasing number of studies have confirmed the important role of SCFAs in the development and progression of lung and other diseases (Qian et al., 2020a; Zhou et al., 2023). For instance, in patients with cystic fibrosis, a disease characterized by thick, sticky mucus in the lungs and other organs, alterations of SCFAs have been found in the airways compared to healthy controls, suggesting a potential role for SCFAs in cystic fibrosis (Miller et al., 2023). Patients with chronic obstructive pulmonary disease have lower levels of SCFAs in their sputum compared to healthy controls (Kotlyarov, 2022). Studies have found that individuals with asthma have altered levels of SCFAs in their airways compared to those without asthma, which may contribute to the chronic inflammation and hypersensitivity seen in the condition (Ito et al., 2023). Furthermore, studies have demonstrated that supplementation of SCFAs shows an initial therapeutic potential in some respiratory diseases. For example, oral supplementation with *Bifidobacterium lactis* help control asthma symptoms (Liu et al., 2021a). Intranasal acetate administration boosts antiviral immunity and reduces virus load during rhinovirus infection (Antunes et al., 2023).

## 5 Evidence supporting the involvement of microbiome and SCFAs in BPD

**Figure 2:**
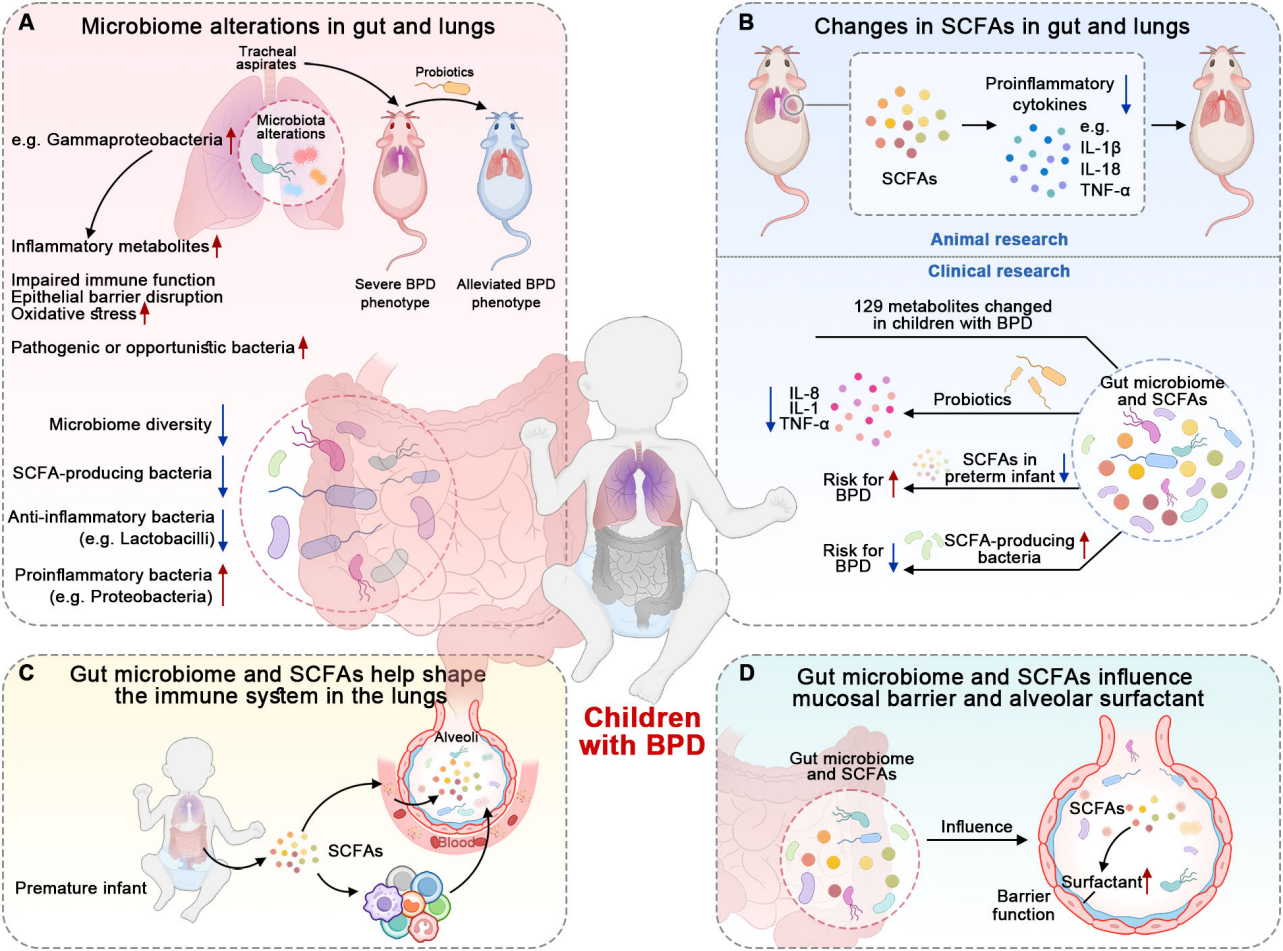
Findings suggesting the roles of microbiome and SCFAs in BPD. **(A)** Infants with BPD have a less diverse microbiome and specific bacteria-dominant dysbiosis. The tracheal aspirates collected from infants with BPD have potential pathogenicity. **(B)** The microbiome’s metabolites, SCFAs, also change in the gut and lungs of children with BPD. SCFAs and probiotics reduce the expression of pro-inflammatory cytokines. **(C)** Microbiome and SCFAs possess the capacity to shape and modulate the immune system. The specific bacteria and SCFAs help restore impaired immune responses. **(D)** Microbiome and SCFAs have the ability to maintain barrier integrity and influence surfactant production, which also suggests their involvement in the pathogenesis of BPD.

There is much evidence supporting the involvement of the microbiome and its metabolites, SCFAs, in the development and progression of BPD. This includes alterations in the microbiome and SCFAs in the gut and lungs, which subsequently affect immune modulation, barrier integrity, and surfactant production (**Figure 2**).

Previous studies have shown that the microbiome is altered in the gut and lungs of children with BPD (**Figure 2A**) (Gallacher et al., 2020; Willis et al., 2023). The gut microbiome in infants who develop BPD is less diverse and less rich in SCFA-producing bacteria compared to healthy controls (Ryan et al., 2019; Zhang et al., 2022). A recent review summarized that preterm infants with BPD tend to have reduced anti-inflammatory bacteria like *Lactobacilli*; at the same time, these infants exhibit an increased abundance of proinflammatory bacteria such as *Proteobacteria* and other potentially pathogenic bacteria (Stricker et al., 2022). These findings suggest a potential link between the gut microbiome, SCFA production, and the development of BPD. In addition to the alteration of the gut microbiome, researchers have also found that the lung microbiome in infants with BPD is also altered (Sun et al., 2021; Stricker et al., 2022). The infants with BPD have *Gammaproteobacteria*-dominant dysbiosis in the lungs, which is linked to worsened BPD outcomes (Freeman et al., 2023). This dysbiosis affects nuclear factor erythroid 2-related factor 2 (NRF2)-mediated antioxidant responses in the lungs, contributing to increased oxidative stress and lung injury severity (Freeman et al., 2023). Moreover, the alterations in the lung microbiome, including microbial structure and function, are observed prior to the onset of BPD in premature infants (Sun et al., 2021). If mice inoculated intranasally with tracheal aspirates from human infants with BPD are exposed to a hyperoxic environment, they exhibit a more severe BPD phenotype than controls; this phenotype can be alleviated by probiotics (Freeman et al., 2023). Even when children with BPD grow to adulthood, their lung microbiome still remains disturbed (Rofael et al., 2019). These studies suggest a causal relationship between the microbiome and BPD.

Beyond the alterations in the microbial composition of the gut and lungs, their metabolites also change in BPD mice (**Figure 2B**) (Zhang et al., 2020; El Saie et al., 2022). For instance, acetate administered by gavage affects lung inflammation and damage in a mouse model of BPD by downregulating the activation of the inflammasomes of NOD-, LRR-, and pyrin domain-containing protein 3 (NLRP3) (Zhang et al., 2020). Acetate treatment was found to reduce the expression of NLRP3-related proteins, such as interleukin-1β (IL-1β) and IL-18, and decrease the levels of inflammatory cytokines, such as tumor necrosis factor-α (TNF-α) (Zhang et al., 2020). This attenuation of the inflammatory response resulted in a reduction in lung injury and improved lung morphology in the BPD mice (Zhang et al., 2020). Moreover, SCFAs can also increase anti-inflammatory cytokines in animal research (Natalini et al., 2023). In terms of clinical studies, there is still a lack of direct evidence supporting the role of SCFAs in children with BPD. However, there are still clinical studies demonstrating the potential roles of SCFAs or other metabolites in BPD. For example, a study found that 129 differentiated metabolites were identified in fecal samples between BPD patients and controls. These metabolites were mainly associated with carbohydrate metabolism (Li et al., 2022). In a cell-based experiment, the probiotics *L. reuteri* and *B. bifidum* reduce the expression of pro-inflammatory cytokines such as IL-8, TNF-α, and IL-1, indicating a possible anti-inflammatory effect (Li et al., 2022). Another example is that preterm infants with decreased levels of SCFAs have a higher risk for BPD (Frazer et al., 2022). On the contrary, infants with a higher abundance of SCFA-producing bacteria in their gut had a lower risk of developing BPD (Stricker et al., 2022). All the animal and clinical findings imply that these SCFAs are involved in the pathogenesis of BPD.

In addition to the changes in the microbiome and SCFAs, other evidence contributing to BPD includes immune modulation (**Figure 2C**), maintenance of barrier integrity, and modulation of surfactant production (**Figure 2D**). They are discussed in other sections.

## 6 Potential mechanisms of microbiome and SCFAs in BPD

**Figure 3:**
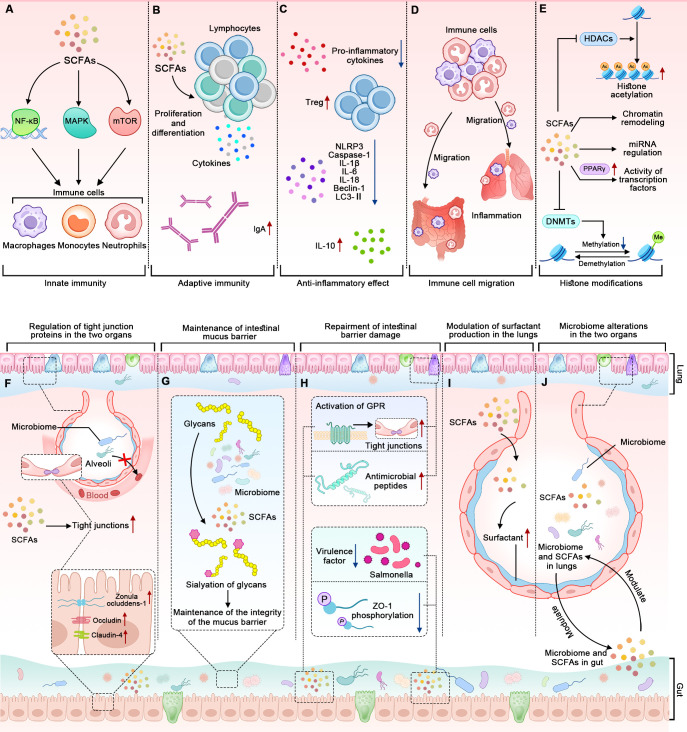
The mechanisms by which alterations in the microbiome and SCFAs contribute to BPD. The panels **(A–D)** represent how the microbiome and SCFAs influence the immune system. **(A)** SCFAs affect innate immune cells through NF-κB, MAPK, and mTOR pathways. **(B)** SCFAs modulate adaptive immune responses. **(C)** SCFAs have anti-inflammatory effects. **(D)** SCFAs influence the migration of immune cells to different tissues. **(E)** SCFAs influence gene expression through epigenetic mechanisms. The panels **(F–H)** represent how the microbiome and SCFAs influence the barrier function of the epithelial cells lining the gut and the lungs. **(F)** The microbiome and SCFAs enhance the expression of tight junction proteins in the two organs. **(G)** Gut microbiome-dependent sialylation of glycans contributes to the integrity of the intestinal mucus barrier. **(H)** SCFAs repair the intestinal barrier by influencing tight junctions, antimicrobial peptides, ZO-1, and virulence factors. The tight junctions and antimicrobial peptides can be observed in the two organs, while the ZO-1 and virulence factors only can be observed in the gut. The panels **(I, J)** show that the microbiome and SCFAs in the gut and lungs interact mutually and modulate surfactant production.

The potential mechanisms of microbiome and SCFAs in BPD are diverse and interconnected, involving various aspects of immune regulation, barrier function, epigenetic regulation, surfactant production, and microbiome alterations (**Figure 3**).

### 6.1 Immune modulation

SCFAs have been shown to influence the immune system in several ways, such as modulating innate and adaptive immunity, exerting anti-inflammatory effects, and influencing the migration of immune cells (**Figures 3A–D**). To highlight the importance of the two latter aspects, we discuss them separately rather than in the context of innate and adaptive immunity.

To begin with, SCFAs significantly impact innate immune cells, including neutrophils, eosinophils, basophils, monocytes, macrophages, and natural killer cells. This occurs through pathways involving nuclear factor-κB (NF-κB), mitogen-activated protein kinases (MAPK), and the mechanistic target of rapamycin (mTOR) (Kim, 2021; Liu et al., 2023). Specifically, SCFAs promote the expansion and functional maturation of innate lymphoid cells (ILCs), including ILC1, ILC2, and ILC3, primarily through their interaction with GPRs like free fatty acid receptor 2. The findings suggest that SCFAs not only enhance ILC responses in the intestines but also in other tissues during infection and inflammation (Sepahi et al., 2021). Another study validated the role of SCFAs on innate immune cells from a therapeutic perspective. Butyrate inhibits lung inflammation by reducing the phagocytic potential of neutrophils and decreasing their extracellular trap formation. This modulation leads to a lower infiltration of neutrophils into lung tissues and a reduction in the release of inflammatory cytokines, thereby alleviating the inflammatory response in the lungs (Sapra et al., 2024).

Moreover, SCFAs play a crucial role in regulating lymphocyte functions by influencing their proliferation, differentiation, and the production of immunoglobulins and cytokines (**Figure 3B**) (Spencer and Bemark, 2023; Mann et al., 2024). Butyrate enhances Foxp3 expression in T cells and increases the stability and functionality of the Foxp3 protein by inhibiting histone deacetylases (HDACs), influencing the balance between pro- and anti-inflammatory immune responses (Arpaia et al., 2013). The gut microbiome and SCFAs can also promote B cell differentiation. SCFAs promote B cell differentiation through several mechanisms. They enhance cellular metabolism by increasing acetyl-CoA levels, which supports energy production and fatty acid synthesis. SCFAs also act as HDACs inhibitors, leading to increased histone acetylation on genes associated with plasma cell differentiation, such as *Aicda* and Ig heavy chain genes. Additionally, SCFAs boost mTOR activation and glycolytic activity, further facilitating B cell activation and differentiation into antibody-secreting cells (Kim et al., 2016). Another study conducted in both humans and mice also revealed that acetate enhances the production of IgA in the colon and alters the IgA pool’s ability to bind to specific microorganisms, including *Enterobacterales* (Takeuchi et al., 2021). These findings illustrate how SCFAs can modulate adaptive immune responses.

Additionally, SCFAs exhibit anti-inflammatory properties (**Figure 3C**). They can inhibit the production of pro-inflammatory cytokines and promote the generation of regulatory T cells, which help maintain immune tolerance and prevent excessive inflammation (Kim, 2021; Schiweck et al., 2022). For example, acetate, propionate, and butyrate can inhibit NLRP3, Caspase-1, IL-1β, IL-6, IL-18, Beclin-1, and microtubule-associated protein 1A/1B-light chain 3-II (LC3-II), and they increase the expression of IL-10 in mice with intestinal mucositis induced by 5-fluorouracil, all of which contribute to maintaining the integrity of the intestinal barrier (Yue et al., 2022; Hirani et al., 2022). The cytokines affected by both microbiome and SCFAs are shown in **Figure 2B**.

Lastly, SCFAs can influence the migration of immune cells to different tissues (**Figure 3D**) (Prado et al., 2023). They attract immune cells, such as neutrophils and macrophages, to inflammatory sites, where they are needed for immune defense. SCFAs can also regulate the migration of immune cells to the gut, promoting immune surveillance and maintaining gut immune homeostasis (Kim, 2021; Liu et al., 2023).

### 6.2 Epigenetic regulation

Recent studies suggest that SCFAs, particularly butyrate, can influence gene expression through epigenetic mechanisms (**Figure 3E**). Butyrate is a potent inhibitor of HDACs, enzymes that remove acetyl groups from histones, leading to a more compact and less accessible chromatin structure (Furusawa et al., 2013; Torow et al., 2023). By inhibiting HDACs, butyrate can promote the acetylation of histones, leading to a more relaxed chromatin structure and enhanced gene expression. SCFAs can also influence DNA methylation patterns by affecting the activity of DNA methyltransferases (DNMTs), the activity of chromatin remodeling complexes, the expression of miRNAs, and the activity of transcription factors (**Figure 3E**) (Jugder et al., 2021; Woo and Alenghat, 2022). However, how these epigenetic regulations influence the pathogenesis of BPD has not been fully elucidated yet.

### 6.3 Enhancement of barrier function

Intestinal barrier integrity is maintained by the surface mucus, epithelial layer, and immune defenses (Camilleri, 2019; Yao et al., 2022; Moonwiriyakit et al., 2023). Animal models of microbiome manipulation support a role for the microbiome in enhancing gut barrier function: germ-free mice exhibit disrupted tight junctions and increased gut permeability, which can be reversed by colonization with a complex microbiome (Kamareddine et al., 2020). In humans, intake of probiotics has been shown to enhance gut barrier function and prevent translocation of gut microbiome into circulation (de Vos et al., 2022). Similarly, the respiratory microbiome and its metabolites also play an important role in maintaining barrier integrity in the lungs (Invernizzi et al., 2020). Overall, microbiome and SCFAs strengthen the barrier function in the gut and/or lungs through at least four ways (**Figures 3F–H**).

One of the ways that microbiome influences the barrier function is to regulate the expression of tight junction proteins (**Figure 3F**). In the gut, they do this by upregulating the expression of tight junction proteins such as occludin, zonula occludens-associated proteins 1 (ZO-1), and claudin-4, which seal the gaps between cells and prevent the translocation of bacteria and toxins (Saleri et al., 2022). In the lungs, SCFAs can strengthen the tight junctions between epithelial cells, preventing the translocation of bacteria and toxins into the lung tissue and the bloodstream (Richards et al., 2020; Karoor et al., 2021).

Another way is the maintenance of the mucus barrier (**Figure 3G**). A study shows that the sialylation of glycans on intestinal mucus, regulated by the microbiome-induced expression of the ST6GALNAC1 enzyme, is essential for maintaining the integrity of the mucus barrier and protecting against excessive bacterial degradation, highlighting the critical role of the microbiome in preserving intestinal barrier function (Yao et al., 2022).

The third way is that SCFAs can repair the intestinal barrier by enhancing expression of tight junction proteins through the activation of GPRs, including GPR41, GPR43, and GPR109A (**Figure 3H**). Perez-Reytor et al. (Perez-Reytor et al., 2021) discussed in detail how the zonula occludens, a toxin produced by *Vibrio cholerae*, disrupts tight junctions by phosphorylating ZO-1 through protein kinase C alpha (PKCα) and how SCFAs may counteract this effect by phosphorylating ZO-1 via PKCβ to restore tight junction assembly. The tight junction proteins are also found in the lungs, and they can be modulated by the microbiome in the gut-lung axis (Alharris et al., 2022).

Other ways to help enhance barrier function include pathogen elimination, anti-inflammation, and providing energy to the cells lining the gut and lungs. For example, intranasal acetate administration showed antiviral effects in a mouse study (Antunes et al., 2023); moreover, this effect was also validated in a separate *in vitro* experiment (Feng et al., 2022). Another study reveals that excessive oxygen exposure in neonatal mice, a frequently used BPD animal model, disrupts antimicrobial peptide expression in the gut; oral supplementation of the antimicrobial peptide helps restore alterations in the intestinal microbiome and alleviate lung injury (**Figure 3H**) (Abdelgawad et al., 2023). Moreover, a study found that SCFAs can inhibit virulence factors of pathogens like *Salmonella* and reduce inflammation caused by toxins in the gut (Perez-Reytor et al., 2021). It is essential to note that more research is needed to fully understand the relationships among the microbiome, SCFAs, and barrier function.

In addition to the above mechanisms, the modulation of surfactant production and the alterations in microbiome themselves are also involvement in the pathogenesis of BPD (**Figures 3I, J**). Although the mechanisms of microbiome and SCFAs in BPD have been studied to some extent, more precise mechanisms remain to be investigated further.

## 7 Treatment implications of microbiome and SCFAs in BPD

The treatment implications of the microbiome and SCFAs in BPD are still being investigated. However, several potential treatment implications have emerged.

Probiotics can help restore a healthy balance of gut microbiome and indirectly influence lung health. Some studies suggest that certain probiotics may have a protective effect against BPD or other respiratory diseases by modulating the immune response, reducing inflammation, and maintaining the barrier integrity of the gut and lungs (Li et al., 2022; Freeman et al., 2023; Sikder et al., 2023; Ngo et al., 2024). Prebiotics are non-digestible fibers that promote the growth of beneficial bacteria in the gut. They can also be used as supplements to influence the microbiome and its metabolites in the gut and lungs, which may be a potential intervention method (Arifuzzaman et al., 2022).

The direct administration of SCFAs has also been shown to be effective in modulating immune responses, maintaining barrier integrity, and attenuating inflammation in the gut and lungs (Antunes et al., 2023; Campbell et al., 2023; Ito et al., 2023). SCFAs can be administered intranasally, in combination with milk, or via drinking water (Antunes et al., 2023; Hildebrand et al., 2023; Ito et al., 2023). However, these studies were mainly conducted in animal models, so there is a need to extrapolate them to clinical settings.

Fecal microbiome transplantation refers to transferring fecal material from a healthy donor to a recipient with an imbalanced gut microbiome. It has been studied in many diseases, such as respiratory diseases (Dong et al., 2024), metabolic diseases (Yang et al., 2024), and intestinal diseases (Bethlehem et al., 2024). The mechanisms by which fecal microbiota transplantation influences lung health likely operate through the gut-lung axis, specifically by modulating gut microbiome diversity, reducing inflammation, and altering immune responses (Lee et al., 2024). SCFAs play a crucial role in these processes (Lee et al., 2024; Verma et al., 2024). However, it has not been studied in BPD.

The use of antibiotics in neonatal intensive care units can disrupt the gut microbiome and increase the risk of dysbiosis. In consideration of the pivotal link between the alterations of microbiome and SCFAs, implementing antibiotic stewardship programs to optimize antibiotic use and minimize unnecessary exposure may help preserve a healthy gut microbiome and reduce the risk of BPD.

## 8 Challenges and future directions

Despite the promising potential of SCFAs in BPD, several challenges need to be addressed. First, the exact mechanisms of how SCFAs modulate lung health and disease are still not fully understood, and more research is needed to elucidate these pathways. Second, while animal studies have shown beneficial effects of SCFAs in lung diseases, clinical studies in humans, especially in premature infants with BPD, are lacking. Therefore, well-designed clinical trials are needed to confirm these findings and determine the optimal dosage and timing of probiotics, prebiotics, and SCFAs (Qian et al., 2020b). Moreover, the gut microbiome is known to be influenced by various factors, including diet, antibiotics, and other medications, which could affect SCFA production. Therefore, strategies to modulate the gut microbiome and SCFA, such as dietary interventions or probiotics, could also be explored as potential treatments for BPD.

## 9 Conclusion

In conclusion, microbiome and SCFAs play a crucial role in the pathogenesis and potential treatment of BPD. Through their anti-inflammatory, immunomodulatory, and barrier-enhancing effects, microbiome and SCFAs could protect against the detrimental effects of BPD. However, further research is needed to fully understand these mechanisms and translate these findings into clinical practice. With more research, microbiome and SCFAs may provide a novel therapeutic approach for BPD, improving outcomes for premature infants affected by this chronic lung disease.
